# Single‐cell transcriptomic atlas of distinct early immune responses induced by SARS‐CoV‐2 Proto or its variants in rhesus monkey

**DOI:** 10.1002/mco2.432

**Published:** 2023-11-20

**Authors:** Yun Yang, Tingfu Du, Wenhai Yu, Yanan Zhou, Chengyun Yang, Dexuan Kuang, Junbin Wang, Cong Tang, Haixuan Wang, Yuan Zhao, Hao Yang, Qing Huang, Daoju Wu, Bai Li, Qiangming Sun, Hongqi Liu, Shuaiyao Lu, Xiaozhong Peng

**Affiliations:** ^1^ National Kunming High‐level Biosafety Primate Research Center, Institute of Medical Biology Chinese Academy of Medical Sciences and Peking Union Medical School Kunming China; ^2^ Key Laboratory of Pathogen Infection Prevention and Control (Peking Union Medical College) Ministry of Education Beijing China; ^3^ State Key Laboratory of Medical Molecular Biology Department of Molecular Biology and Biochemistry Institute of Basic Medical Sciences Medical Primate Research Center Neuroscience Center Chinese Academy of Medical Sciences School of Basic Medicine Peking Union Medical College Beijing China

**Keywords:** COVID‐19, immune response, nonhuman primate, PBMCs, SARS‐CoV‐2, single‐cell sequencing

## Abstract

Immune responses induced by severe acute respiratory syndrome coronavirus 2 (SARS‐CoV‐2) infection play a critical role in the pathogenesis and outcome of coronavirus disease 2019 (COVID‐19). However, the dynamic profile of immune responses postinfection by SARS‐CoV‐2 variants of concern (VOC) is not fully understood. In this study, peripheral blood mononuclear cells single‐cell sequencing was performed to determine dynamic profiles of immune response to Prototype, Alpha, Beta, and Delta in a rhesus monkey model. Overall, all strains induced dramatic changes in both cellular subpopulations and gene expression levels at 1 day postinfection (dpi), which associated function including adaptive immune response, innate immunity, and IFN response. COVID‐19‐related genes revealed different gene profiles at 1 dpi among the four SARS‐CoV‐2 strains, including genes reported in COVID‐19 patients with increased risk of autoimmune disease and rheumatic diseases. Delta‐infected animal showed inhibition of translation pathway. B cells, T cells, and monocytes showed much commonality rather than specificity among the four strains. Monocytes were the major responders to SARS‐CoV‐2 infection, and the response lasted longer in Alpha than the other strains. Thus, this study reveals the early immune responses induced by SARS‐CoV‐2 Proto or its variants in nonhuman primates, which is important information for controlling rapidly evolving viruses.

## Graphical abstract

Infection of SARS‐CoV‐2 (Prototype, Alpha, Beta, Delta) induced dramatic immune response in peripheral blood mononuclear cells (PBMCs). Prototype caused stronger immune response and IFN response. And the Delta strain has more effect on the translation pathway. Four strains caused monocytes as major responder of the PBMCs the on 1 day postinfection, and increased risk of autoimmune disease and rheumatic diseases.

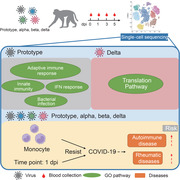

## 1 INTRODUCTION

With its high transmission rate, rapid evolution, and scope of influence, the severe acute respiratory syndrome coronavirus 2 (SARS‐CoV‐2) virus, first reported in December 2019, has caused an unprecedented pandemic.^1^ The ongoing coronavirus disease 2019 (COVID‐19) pandemic poses a huge challenges to its global prevention and control.^2^ Derived from the prototypic strain (Proto), more than dozens of SARS‐CoV‐2 variants have emerged, including four variants of concern (VOCs): Alpha, Beta, Delta, and Omicron.^3^ The emergence of each VOC has aroused great concern worldwide because of its distinct biological characteristics according to clinical or laboratory studies, particularly the increased transmission, immune escape from previous vaccines, and the more severe disease.^4^ The emerging variants or subvariants of SARS‐CoV‐2, particularly Omicron, are reinfecting some of the vaccinated populations around the world.^5^ Emerging evidence from post‐COVID‐19 studies has highlighted distinct patient profiles upon infection with different strains, implying variations in the mechanistic aspects of variant infections.^6^, ^7^

Many studies have been conducted to explore SARS‐CoV‐2 etiology, including viral gene and protein functions,^8^, ^9^ structural biology of virus,^10^ and interactions between virus and host cells.^11^ However, studies on host responses to SARS‐CoV‐2 infection are insufficient to fully elucidate some critical questions, particularly the immunological mechanism of the host. Immune responses play an important role in preventing SARS‐CoV‐2 infection and in rehabilitating COVID‐19. However, dysfunction of immune regulation may result in immunopathological damage, such as a cytokine storm.^12^ Among the VOCs, there are distinct differences in immunological characteristics, such as the degree of immune escape and cross‐neutralization,^11^ but more studies are urgently needed to explore the mechanisms. Because of the limitations in the clinical studies, some clinical results still need to be verified in animal models, especially in a nonhuman primate (NHP) model that is physiologically close to human beings. For example, it is impossible to precisely determine the infection time point of COVID‐19 patients, and ethics strictly limits sampling for analysis of histopathological lesions. In the past 3 years, NHP models of SARS‐CoV‐2 infection have been extensively used and made great contributions to elucidate the pathogenesis of SARS‐CoV‐2 and develop both vaccines and drugs.^13^ However, the underlying dynamic immune response mechanisms have not been fully elucidated.

Single‐cell RNA sequencing (scRNA‐seq) is an ideal tool for studying comprehensively host responses to SARS‐CoV‐2 infection because of the power of high‐resolution capability.^14^ Reports samples for scRNA‐seq are involved in lung tissues,^15^ bronchoalveolar lavage,^16^ peripheral blood mononuclear cells (PBMCs),^17^, ^18^, ^19^ intestinal tissues, and lymphoid tissues (spleen, lymph nodes).^20^ PBMCs have several advantages over other tissues for immunological studies. First, it is easy to obtain PBMCs from patients with COVID‐19 in hospital or animal infected with SARS‐CoV‐2. Furthermore, PBMCs contain circulating immune cells that can reflect systemic immune responses. Several groups have revealed the landscape of immunological responses in COVID‐19 patients via scRNA‐seq.^14^ Most cells in PBMCs from COVID‐19 patients show strong interferon‐α and acute inflammatory responses.^18^ The severity of COVID‐19 positively correlates with the heterogeneity of interferon stimulation gene (*ISG*) signature and profound immune exhaust. Massive expansion of highly cytotoxic T‐cell subsets is associated with convalescence in moderate disease patients.^19^

Nevertheless, to our knowledge, there is few comparative reports of immune responses induced by SARS‐CoV‐2 Proto and its variants since it is hard to conduct this kind of study in COVID‐19 patients. In this study, based on our previously established rhesus monkey model of SARS‐CoV‐2 infection, we analyzed the dynamic immune profile of PBMCs post‐SARS‐CoV‐2 or its VOC infection via scRNA‐seq technology. All SARS‐CoV‐2 strains induced an early immune response at 1 day postinfection (dpi), and distinct cell lineages and gene profiles were observed among Proto and SARS‐CoV‐2 VOCs. This study further revealed that the upregulation of some genes is probably associated with an increased risk of autoimmune disease and rheumatic diseases. These results may be beneficial in explaining the clinical features and sequelae of COVID‐19 patients.

## 2 RESULTS

### 2.1 Dramatic change in immune cell lineages in PBMCs early post‐SARS‐CoV‐2 infection

**FIGURE 1:**
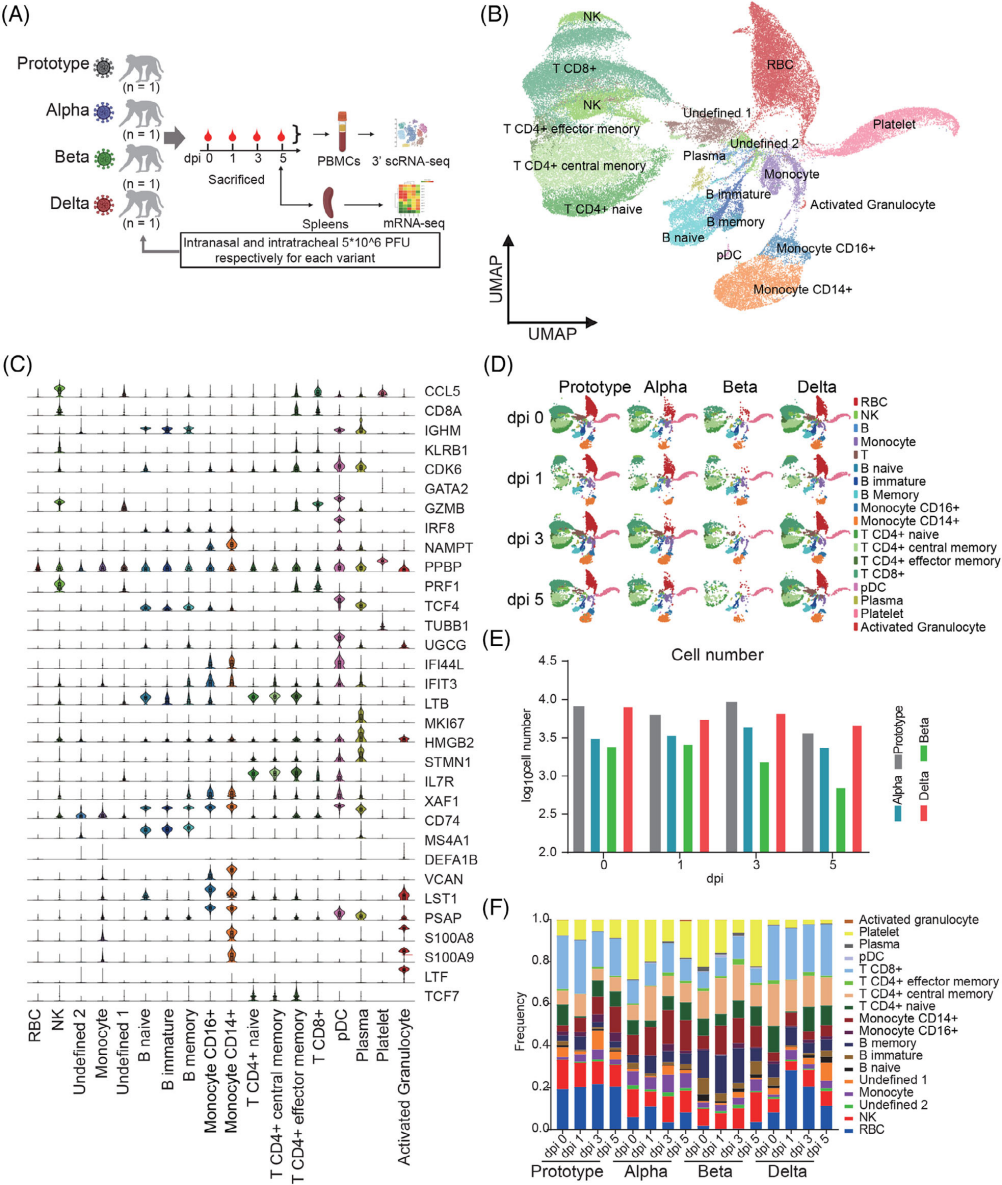
Single‐cell sequencing landscape of PBMCs in rhesus monkeys infected with SARS‐CoV‐2 Proto or its variants. (A) Schematic representation of experimental design. Rhesus monkeys were inoculated with Prototype, Alpha, Beta, or Delta SARS‐CoV‐2 strains via intranasal (0.5 mL) and intratracheal (0.5 mL) routes at 10^7^ pfu/ml doses. Samples were collected at the indicated time points for further analysis. (Figure was created with BioRender.com.) (B) UMAP dimensionality of PBMCs from all profiled samples (*n* = 73 453 cells). R package SingleR was used to annotate cell types based on the Human Cell Landscape database and manual cell‐type annotation. (C) Expression levels of selected canonical cell markers in cell lineages. (D) UMAP of major immune cells in PBMCs. Each sample is colored by cell types. (E) Cell number in each PBMC sample of four monkeys infected with SARS‐CoV‐2 Proto or its variants at 1, 3, and 5 days postinfection (dpi). (F) Frequencies of cell lineages in PBMCs of each sample.

The NHP model of SARS‐COV‐2 infection has been widely used for studying COVID‐19 pathogenesis and antiviral research because of its unique features.^21^ Using this model, we comprehensively analyzed the dynamic immune profile of PBMCs postinfection with SARS‐CoV‐2 Proto or its variants via scRNA‐seq (Figure 1A). We first confirmed viral presence in swab and lung samples via quantitative reverse transcription PCR (RT‐qPCR), and then we detected viral nucleoprotein (*N*) and analyzed the histopathology in the lung tissue via immunofluorescence staining and HE staining, respectively. The results suggested successful infections and diseases caused by the Proto virus or its variants (Figures S1A and B). However, we had not observed clinical symptoms in infected monkeys, consistent with previously reported animal models.^22^, ^23^ Then, scRNA‐seq was performed to analyze the immune profile of 16 PBMC samples collected at 0, 1, 3, and 5 dpi. In this experiment, a total of 73,453 cells were analyzed through 3′ single‐cell sequencing. According to specific gene markers, PBMCs were clustered into 18 distinct subpopulations, including NK, CD8+ T, CD4+ T effector memory, CD4+ T central memory, CD4+ T naïve cells, plasma, other B, B immature, B memory, B naïve, pDC, other T cells, monocytes, activated granulocytes, platelets, and red blood cells (RBCs) (Figures 1B and C). The comparison between 16 samples revealed that the cell lineages of the samples collected at 1 dpi were different from those at 0, 3, and 5 dpi, mainly NK cells, CD4+ T central memory cells, B memory cells, CD8+ T cells, plasma cells, and CD14+ monocytes (Figure 1D). After filtering out low‐quality cells, we obtained transcriptome data sets from 73,153 cells (Figure 1E). The frequencies of several cell lines decreased dramatically at 1 dpi and then returned to normal level at 3 dpi (except Beta), such as B naïve, B immature, CD16, NK, and CD4+ T naïve cells. On the contrary, the frequencies of B memory, CD8+ T, CD4+ T central, and monocyte increased at 1 dpi and returned to normal level at 3 dpi (except Beta) (Figure 1F). Last, we found low expression levels of *ACE2* and *TMPRSS2*, critical factors in SARS‐CoV‐2 infection, and no viral RNA detectable in PBMCs (Figure S1C), consistent with results of clinical studies in COVID‐19 patients. These results suggest that SARS‐CoV‐2 or VOC infection leads to dramatic changes in immune cell lineages in PBMCs.

### 2.2 Different gene profiles of PBMCs from animals infected with SARS‐CoV‐2 Proto or its variants

**FIGURE 2:**
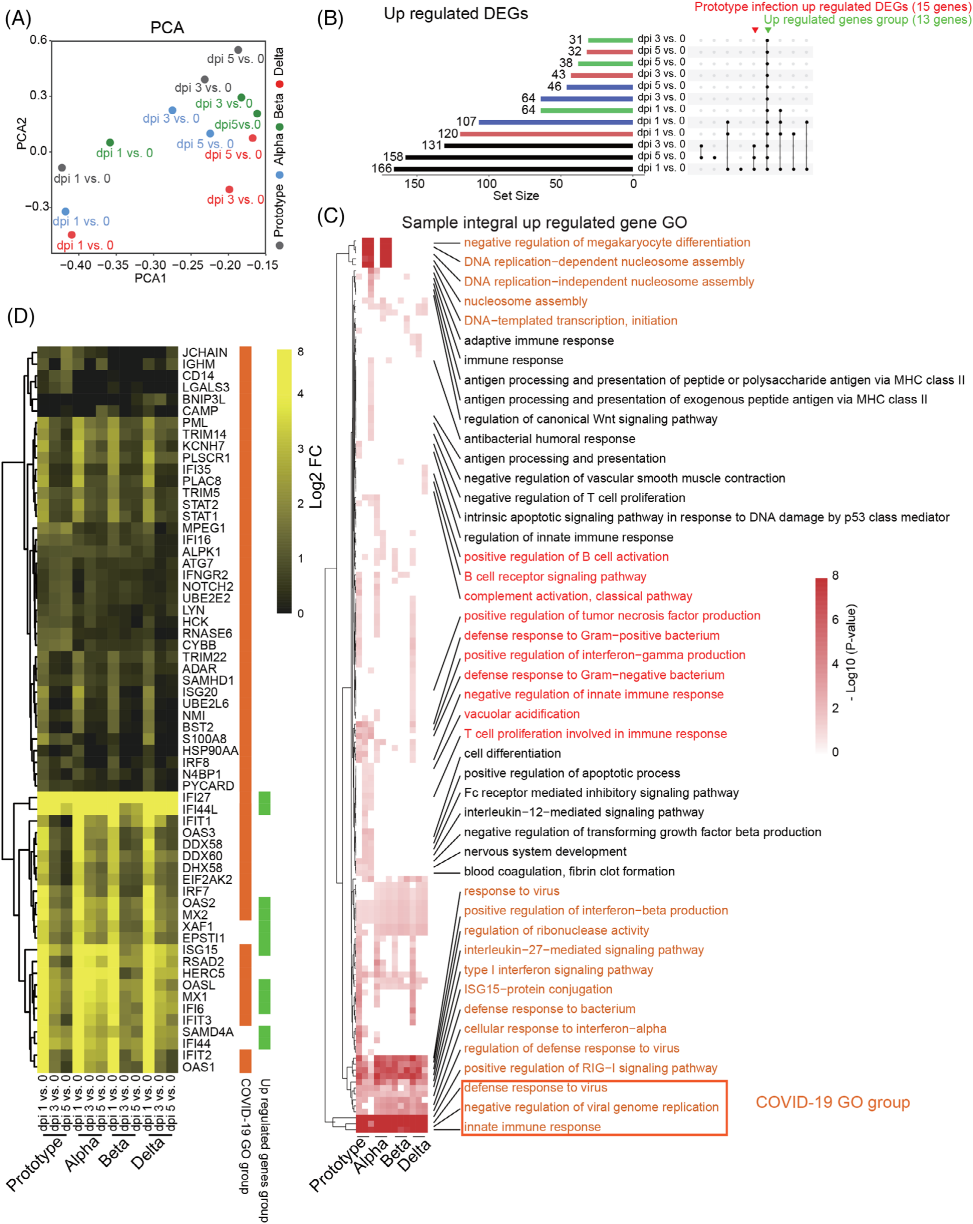
Integral responses of PBMCs to infection by SARS‐CoV‐2 Proto or its variants in rhesus monkeys. (A) PCA was performed on differentially expressed genes (DEGs) (*p* < 0.05, log2 FC > 1, −log2 FC < −1) of PBMCs collected at 1, 3, or 5 dpi vs. 0 dpi via R package ggplot2. Dots are colored by variants. (B) UpSet plots of integral up‐regulated DEGs of PBMCs collected at 1, 3, or 5 dpi vs. 0 dpi. The set size bar is colored by variants using the R package UpSetR: Prototype (gray), Alpha (blue), Beta (green), and Delta (red). (C) Upregulated DEGs enriched gene ontology (GO) pathways. (D) DEGs of COVID‐19‐related genes in PBMCs collected at 1, 3, or 5 dpi vs. 0 dpi.

**FIGURE 3:**
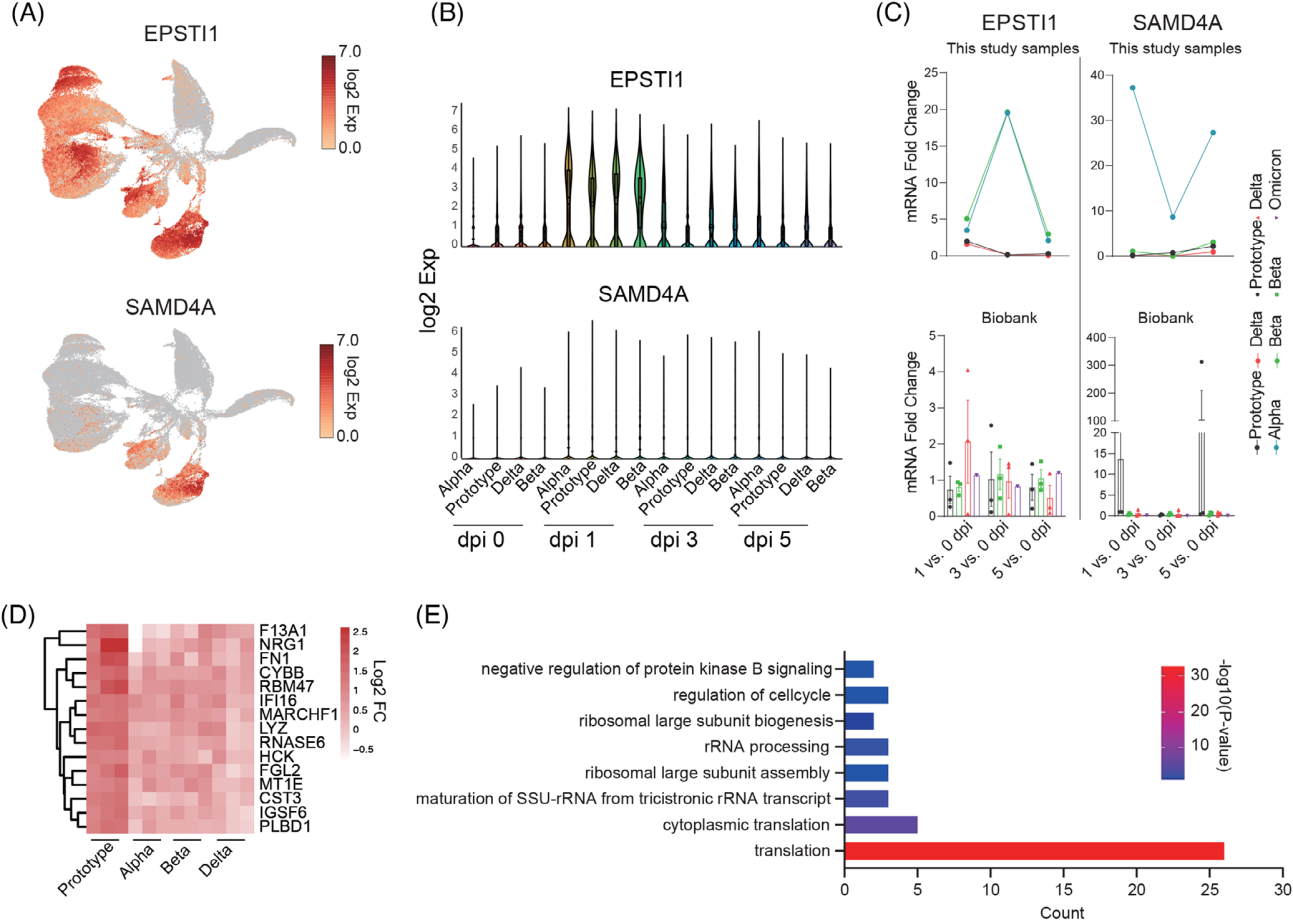
Differences in rhesus monkey PBMC infection with SARS‐CoV‐2 Proto or its variants. (A) Expression of *EPSTI1* and *SAMD4A* in PBMC lineages. The heatmap is produced in UMAP. (B) Expression of *EPSTI1* and *SAMD4A* in PBMCs collected at 0, 1, 3, or 5 dpi. (C) Quantitative reverse transcription PCR (RT‐qPCR) of *EPSTI1* and *SAMD4A* expression in the PBMC samples of this study (Prototype, *n* = 1; Alpha, *n* = 1; Beta, *n* = 1; and Delta, *n* = 1) and biobank PBMC samples (Prototype, *n* = 3; Beta, *n* = 3; Delta, *n* = 3; and Omicron, *n* = 1); data are presented as mean ± s.e. (D) Significant upregulation of genes in PBMCs collected at 1, 3, or 5 days postinfection with Proto vs. 0 dpi. (E) GO analysis of downregulated DEGs at 1 day post‐Delta infection.

Next, principal component analysis (PCA) of differentially expressed genes (DEGs) post‐scRNA‐seq was conducted to analyze all genes of 16 PBMC samples from rhesus monkeys infected with SARS‐CoV‐2 Proto or its variants. We used samples collected at 0 dpi (before infection) as negative controls to eliminate individual heterogeneity. DEGs at 1 dpi with all four SARS‐CoV‐2 strains were clustered separately from those at 3 and 5 dpi (Figure 2A). The DEGs post‐SARS‐CoV‐2 infection mostly belonged to upregulated genes, few of which were downregulated genes. Thirteen upregulated gene clusters (for convenience, these gene clusters were referred to as upregulated gene groups) were observed at 1, 3, and 5 dpi with Proto or its variants. However, the genes upregulated in PBMCs were mostly from Proto‐infected animals and were observed at various times. Among the four SARS‐CoV‐2 strains tested, infection of the Delta strain induced relatively more downregulated genes at 1 dpi (Figures 2B and S1D). We then examined the pathways in which upregulated genes (sample integral upregulated gene in gene ontology (GO)) were involved (Figure 2C). The pathways that were highly related to four strains of SARS‐CoV‐2 infection were mainly viral inhibition and innate immunity‐associated pathways, including the type I interferon signaling pathway, cellular response to interferon‐alpha, IL‐27‐mediated signaling pathway, conjugation of ISG15 protein, and positive regulation of the RIG‐I signaling pathway. Additionally, the pathways that are particularly involved in Proto and Alpha strain infections were mainly associated with megakaryocyte (MK) and DNA repair, which converged on the histone H4 of the gene. The upregulated genes in PBMCs of Proto strain‐infected animals were related to some unique GO pathways, including antigen processing and presentation, Wnt pathway, and immune cell‐associated pathways. Notably, some upregulated DEGs were involved in the defense response to bacteria during Proto strain infection. Delta strain infection upregulated DEGs enriched in pathways of B cell activation and B receptors (Figure 2C). Expression levels of some COVID‐19‐related genes peaked at 1 dpi and then decreased (particularly for the Proto strain), including macrophage‐associated genes (*OASL, OAS1‐3, IFIT1‐3, ISG15*, and *HERC5*) and type I interferon‐associated genes (*IFI44, IFI44L, RSAD2*, and *MX1‐2*) (Figure 2D). Interestingly, among upregulated genes, two genes (EPSTI1 and SAMD4A) have rarely been reported in COVID‐19‐related group genes and were not associated with any related signaling pathway. *EPSTI1* is expressed in all populations of immune cells and peaked at 1 dpi, while *SAMD4A* is expressed mainly in B cells and monocytes (Figures 3A and B). We examined via RT‐qPCR the expression levels of the COVID‐19‐related group genes in PBMCs of four monkeys from this study infected by Prototype, Alpha, Beta, or Delta, as well as other rhesus monkey samples (Prototype, *n* = 3; Beta, *n* = 3; Delta, *n* = 3; and Omicron, *n* = 1) from biobank (Figures S2, S3 and S4). *EPSTI1* and *SAMD4A* were found to be upregulated in all SARS‐COV‐2‐infected animals of this study, consistent with the single‐cell sequencing data (Figure 3C). Among the upregulated genes that are associated with SARS‐CoV‐2 infection (Figure 2B), 15 were related to adaptive immunity, innate immunity (*LYZ*, coding for lysozyme), interferon response, and bacterial infection, which was particularly noticeable in the PBMCs of Proto‐infected animal (Figure 3D). The downregulated DEGs appeared in samples mainly at 1 day post‐Delta strain infection (Figure S1D) and were mainly enriched in the translation pathway of RPL and RPS family (Figure 3E). These results indicate that SARS‐CoV‐2 Proto and its variants caused distinct gene profiles of PBMCs in rhesus monkeys.

### 2.3 B cells, T cells, and monocytes respond differentially to infection of SARS‐CoV‐2 Proto or its variants

**FIGURE 4:**
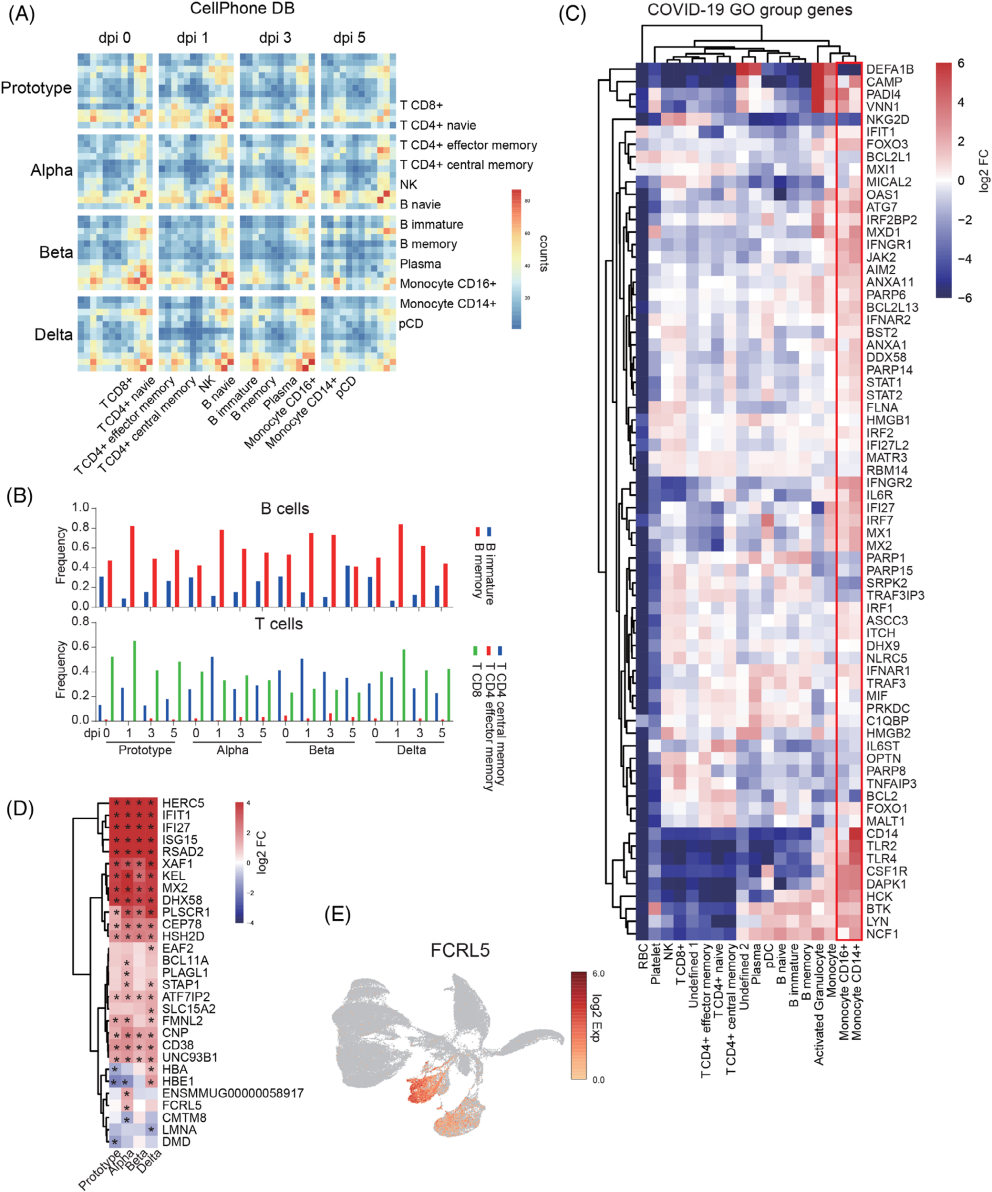
Monocytes in PBMCs are the main cell type involved in the immune response to infections by SARS‐CoV‐2 Proto or its variants. (A) Cell–cell communications of main cell lineages in PBMCs collected at 0, 1, 3, and 5 dpi with SARS‐CoV‐2 Proto or its variants. (B) Frequencies of B cells, and T cells in PBMCs collected at 0, 1, 3, and 5 dpi. (C) Expression of COVID‐19 GO group genes (GO: 0051607, GO: 0045087, and GO: 0045071) in PBMC lineages. (D) Expression of DGEs (*p* < 0.05) in B memory cells of PBMCs at 1 dpi (vs. 0 dpi). (E) Expression of *FCRL5* in cell lineages of PBMCs at 1 dpi.

Based on scRNA‐seq data, cell–cell communication was further predicted. Overall, cellular communication in PBMCs increased at 1 dpi with SARS‐CoV‐2 Proto or its variants, particularly communications among monocytes or between monocytes and CD4+ T cells. However, cellular communication in PBMCs of Proto‐ and Beta‐infected animals decreased rapidly at 3 dpi. In contrast, cellular communication in the Alpha and Delta strain PBMCs lasted longer than that in the Prototype and Beta strains. PBMCs after Alpha strain infection showed the strongest CD14 and CD16 monocyte communication among the four strains. PBMCs in the Delta strain showed the strongest communication between CD14 monocytes and pDC among the four strains (Figure 4A).

Three major lineages of immune cells changed post‐SARS‐CoV‐2 infection, particularly at 1 dpi, namely B cells, T cells, and monocytes. B memory showed high frequency in PBMCs at 1 and 3 days post‐Beta strain infection. CD4+ T central memory cells showed a high frequency in PBMCs at 1 dpi post‐Alpha or ‐Beta infection. CD8+ T cells showed high frequency in PBMCs at 1 dpi with Prototype or Delta strain (Figures 4B and S1E). Analysis of DEG enrichment in three important SARS‐CoV‐2 pathways (defense response to virus, innate immune response, and negative regulation of viral genome replication) showed that monocytes were the major responders to SARS‐CoV‐2 infection (Figure 4C).

As suggested by the results above, PBMCs at 1 dpi showed the strongest responses to four strains of SARS‐CoV‐2. Therefore, we focused on a horizontal comparison of PBMCs among four strains at 1 dpi. First, we examined DEGs in subpopulations of immune cells in PBMCs. The results showed that CD14+ cells possessed the highest DEGs, followed by CD8+ T cells, CD4+ T central memory cells, and plasma cells (Figure S1F). However, B memory cells formed two clusters according to DEGs (Figure 4D). In one cluster, B memory cells showed highly upregulated DEGs but without difference among four strains, including *HERC5, IFIT1, IFI27, ISG15*, and *RSAD2*. In the other cluster, B memory cells showed several DEGs among four strains. Interestingly, *FCRL5*, which is associated with the function of B memory cells, was highly upregulated at 1 day post‐Alpha or ‐Delta infection in PBMCs compared with those in PBMCs post‐Proto or ‐Beta infection (Figure 4D). In addition, *FCRL5* was expressed in monocytes (Figure 4E). Finally, plasma cells also showed great differences in DEGs between four viral strains, similar to the published data.^24^ Furthermore, plasma cells in PBMCs of Proto‐infected animals had the most upregulated genes (27 out of 50), suggesting that the Proto strain infection was distinct from other viral strains. *IGHM* (IgM coding gene) and *AIRE* (IL12 associated gene) were also highly upregulated in PBMCs at 1 day post‐Proto, ‐Alpha, or ‐Delta infection. Furthermore, compared with the other strains of SARS‐CoV‐2, the Alpha strain induced higher levels of *ASPM*, *CENPF*, and *CCDC134*. Upregulation of genes in Beta strain PBMCs was not obvious, indicating that the Beta strain infection induced relatively weak immune responses (Figure S1G). These results indicate that B cells, T cells, and monocytes respond differentially to infection with SARS‐CoV‐2 Proto or its variants.

### 2.4 Long‐lasting responses of monocytes in PBMCs to SARS‐CoV‐2 infections

**FIGURE 5:**
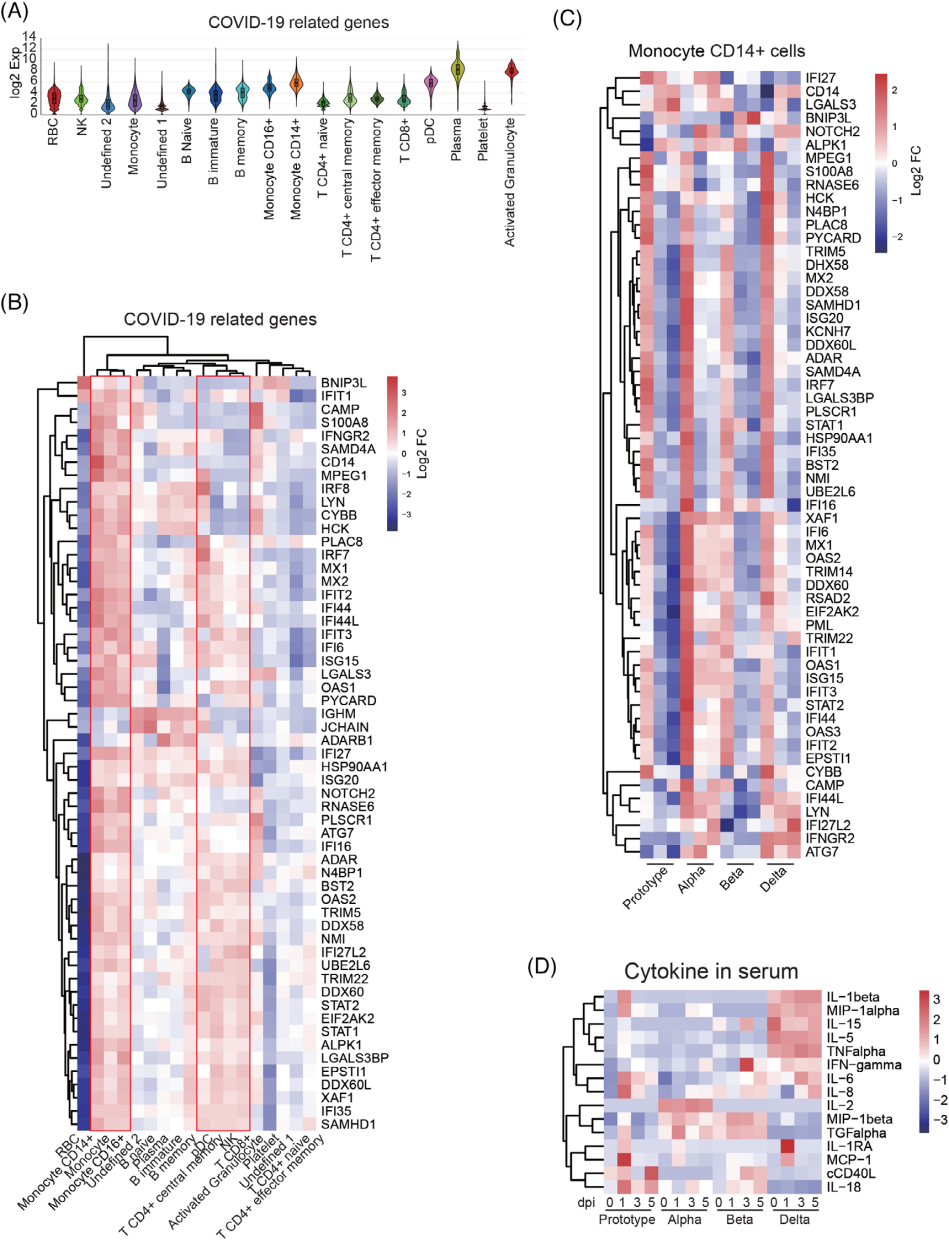
CD14+ or CD16+ monocytes in PBMCs show long‐standing responses to SARS‐CoV‐2 infections. (A) Distributions of COVID‐19 related genes in PBMC lineages. (B) Expression levels of COVID‐19‐related genes in PBMC lineages. (C) Expression levels of COVID‐19‐related genes in CD14+ monocytes from PBMCs collected at 1, 3, and 5 dpi (vs. 0 dpi). (D) Levels of cytokines in serum samples collected at 0, 1, 3, and 5 dpi. The data were normalized in each row.

As shown in Figure 2D, we found a list of genes in rhesus monkeys that were highly related to SARS‐CoV‐2 infection and mostly enriched in plasma cells, followed by pDC, activated granulocytes, CD14+ cells, and CD16+ cells (Figure 5A). When we examined the expression of specific genes for subpopulations of immune cells, we found that most genes were upregulated in monocytes, T cells, NK cells, and pDCs, suggesting that they were the main responders to SARS‐CoV‐2 infection. In addition, some genes in B cells were upregulated in response to infection, including *IGHM*, *JACHAIN*, and *ADARB1* (Figure 5B). Among these responding cell populations, monocytes were the strongest responders to infection, followed by CD4+ T central memory, NK, and CD8+ T cells. The other subpopulations of immune cells had low frequency, which resulted in high DEGs. In fact, these cells did not actively respond to infection. Therefore, we next examined the dynamics of SARS‐CoV‐2‐related genes in monocytes of PBMCs from four strains of virus‐infected animals. In CD14+ cells (Figure 5C), most of these genes were sharply upregulated at 1 dpi with any of the four strains of the virus and then decreased at 3 and 5 dpi. However, some genes still maintained high levels at 3 and 5 dpi, including *IFI16*, *XAF1*, *IFI6*, *MX1*, *OAS2*, *TRIM14*, *DDX60*, *RSAD2*, *EIF2AK2*, *PML*, *TRIM22*, *IFIT1*, *OAS1*, *ISG15*, *IFIT3*, *STAT2*, *IFI44*, *OAS3*, *IFIT2*, and *EPSTI1* in CD14+ cells from animal infected with the Alpha strain. Similarly, in CD16+ cells (Figure S5A), the Alpha strain infection induced continuous upregulation of *IFI16*, *IFI27*, *NOTCH2*, *LGALS3*, *KCNH7*, *IFIT2*, *ADAR*, *IFIT3*, *HCK*, *TRIM5* and *STAT2*, and *ALPK1*, *TRIM22*, *STAT1*, *EPSTI1*, *OAS2*, *PML*, and *IFI44* were continuously upregulated in Beta strain‐infected animal. SARS‐CoV‐2‐related genes in other subpopulations (e.g., NK, CD4+ T central memory cells, and CD8+ T cells) of immune cells consistently showed high expression at 1 dpi and downregulation at 3 and 5 dpi (Figure S6). The SARS‐CoV‐2‐related genes in B memory cells showed no obvious differences among the four viral strains (Figure S5C). Notably, B memory cells from the four strains of viruses showed continuous and high expression levels of *IFI27*. Furthermore, *IL15RA* was highly expressed in B memory cells at 1 dpi with any of the viral strains. In contrast, *IL1B* was always expressed low during the experiment among four strains. Finally, we analyzed the expression of inflammation‐related genes in a subpopulation of immune cells in PBMCs based on the gene list from *GO0006954* inflammatory response. Monocytes, particularly CD14+ cells, played an important role in inflammatory responses (Figure S5B). At the protein level, infection with SARS‐CoV‐2 Proto or its variants reduced some inflammatory cytokines, but there was no obvious difference between the four viral strains (Figure 5D). These results suggest that monocytes in PBMCs are the long‐lasting responders to SARS‐CoV‐2 infections.

### 2.5 SARS‐CoV‐2 Proto induces transcriptional responses in the spleen distinct from those of its variants

**FIGURE 6:**
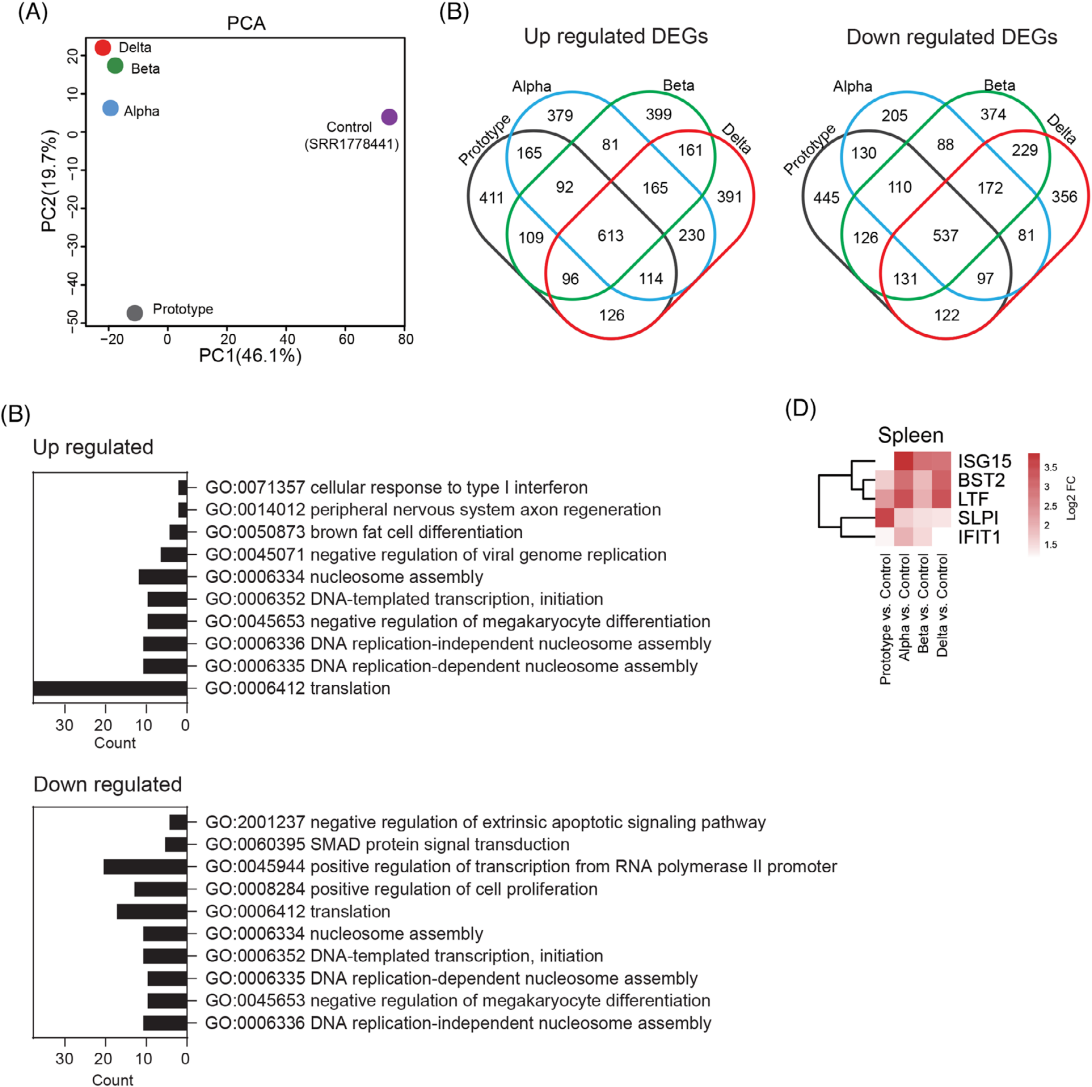
Transcriptomic profile of spleen postinfection with SARS‐CoV‐2 Proto or its variants. Differentially expressed genes (DEGs) were determined with *p* < 0.05, log2 FC > 1, −log2 FC < −1 in infected groups vs. noninfected control with reference number SRR1778441. (A) PCA was performed on DEGs of spleens collected at 5 dpi via R package ggplot2. (B) Venn plots were utilized to show upregulated and downregulated genes in spleens postinfection with SARS‐CoV‐2 Proto or its variants. (C) Major GO pathways (*p* < 0.05) enriched with upregulated or downregulated genes in the spleen. (D) Expression of genes enriched in negative regulation of viral genome replication pathway (GO:0045071) postinfections vs. noninfected control with reference number SRR1778441.

The spleen has been documented to be one of the important extrapulmonary targets of COVID‐19.^25^, ^26^ Therefore, we examined the transcriptome of the spleen post‐infection with SARS‐CoV‐2 Proto or its variants, and data from the SRA database (SRR1778441) for uninfected monkeys were used as negative control. The PCA of the transcriptomic data showed that at 5 dpi there was a slight dispersion but no separation (Figure 6A), which is consistent with the result of the scRNA‐seq (Figure 2A). Further DEG analysis revealed that SARS‐CoV‐2 infection led to spleen transcriptional changes (upregulation and downregulation), including some strain‐specific regulation of genes (Figure 6B). Functional analysis of upregulated genes in all viral strains indicated that they were associated with translation pathways, DNA replication, RNA splicing, energy metabolism, and negative regulation of MK differentiation (Figures 6C and S7), suggesting differentiation of a large number of immune cells post‐SARS‐CoV‐2 infection. Comparison among viral strains showed that genes in the spleens of Proto‐ or Alpha‐infected animals were mainly enriched in the inflammatory response. In contrast, genes in the spleens of Delta‐infected animals were mainly enriched in viral process and adaptive immune response, and those in Beta enriched in positive regulation of gene expression. Downregulated genes in the spleens were mainly enriched in RNA polymerase‐associated pathways (Figures 6C and S7). Consistent with the results from PBMCs, *ISG15* was highly expressed in Alpha, Beta, and Delta strain‐infected spleens but not in the Proto strain‐infected spleen (Figure 6D). These results suggest that SARS‐CoV‐2 Proto induces transcriptional responses in the spleen distinct from those of its variants.

## 3 DISCUSSION

Since the first report of SARS‐CoV‐2, the virus has been rapidly evolving, leading to variants.^27^ The immune‐related characteristics of these variants attract global attention because understanding these characteristics will be beneficial in preventing and controlling COVID‐19. Mutations in the S protein of variants, particularly those of VOCs, usually cause immune escape from protection induced by the Proto virus.^11^ SARS‐CoV‐2 infection activates the immune system to induce cytokine storm, which is widely recognized as an important factor in the pathogenesis of COVID‐19.^12^ Comparison of emerging variants with the previous strain can only be performed retrospectively because it is rare that there are two different variants of SARS‐CoV‐2 circulating in the same region. Using an established NHP model of SARS‐CoV‐2 infection, in this study, we infected rhesus monkeys with Proto SARS‐CoV‐2 or its VOCs. We have analyzed the dynamic profile of immune responses in PBMCs postinfection with SARS‐CoV‐2 Proto or its variants at the single‐cell level, revealing distinct early immune responses induced by SARS‐CoV‐2 Proto or its variants in NHPs.

Although this is an animal study of SARS‐CoV‐2 infection, there are at least three advantages over clinical studies of COVID‐19. First, it has been reported that most severe or critical COVID‐19 patients have comorbidities, and infection of SARS‐CoV‐2 may be different, affecting their immune status. In our study, we screened animals before the experiment to minimize the effects of other factors. Second, it is impossible to find if SARS‐CoV‐2 Proto or its variants infected patients simultaneously to study the immune response. However, an animal study allows us to study SARS‐CoV‐2 Proto and its variants simultaneously and under the same conditions. Third, a dynamic analysis of immune responses to COVID‐19 has been conducted in two ways, one based on the severity of COVID‐19 (mild, moderate, and severe)^27^ and the other based on precise time points of sample collection.^28^ However, during sampling, COVID‐19 patients may be treated in various ways. Therefore, it is theoretically impossible to exclude the influence of other factors. In this study, we could keep experimental animals under the same conditions during sampling. Finally, NHPs are physiologically close to human beings. Therefore, the results of this study are easily translated into the clinical prevention of COVID‐19.

Early immune responses, particularly innate immune responses, play critical roles in controlling the occurrence and development of infection. In this study, we analyzed dynamic immune responses of PBMCs post‐SARS‐CoV‐2 infection via scRNA‐seq. Overall, strong immune responses occurred at 1 dpi and then decreased in the following days, including the frequencies of immune cell populations (Figures 1E and F), COVID‐19‐related genes and signal pathways (Figures 2 and 3), COVID‐19‐related genes intersected with a study including a very large patient samples, and 16 genes (*IFI27*, *IFI44L*, *IFIT1*, *OAS3*, *DDX60*, *OAS2*, *XAF1*, *ISG15*, *RSAD2*, *HERC5*, *MX1*, *IFI6*, *IFIT3*, *IFI44*, *IFIT2*, and *OAS1*) overlapped with them, suggesting that rhesus monkeys can mimic human infection with SARS‐CoV‐2.^29^ Furthermore, strong interferon‐α/interferon‐β response indicates strong antigen‐presenting and antiviral CD8+ T cell responses.^30^ In particular, upregulation of *EPSTI1* and *SAMD4A* was observed in all SARS‐CoV‐2 strains tested in this study, including Omicron. *EPSTI1* was enriched in all populations of immune cells, while *SAMD4A* was enriched mainly in B cells and monocytes (Figures 2 and 3). *EPSTI1* was highly expressed in peripheral blood and B cells in labial gland tissue from primary Sjogren syndrome. *EPSTI1* regulates B cell proliferation and activation through the NF‐KB pathway, resulting in autoimmune disease.^31^ Upregulation of *SAMD4A* is possibly associated with an increased risk of rheumatic disease post‐COVID‐19.^32^ Therefore, in the current NHP mode, it will be significant and interesting to investigate rheumatic disease after SARS‐CoV‐2 infection, particularly the Alpha variant. *XAF1*, which is involved in the host's response to RNA virus,^33^ was upregulated at 1 dpi. Similar early immune responses were also observed in the spleen (Figure 6 and S7). In PMBCs, monocytes, B cells, and plasma cells were the main responders to SARS‐CoV‐2 infection (Figures 4 and S1), consistent with the results reported in clinical COVID‐19 patients.^17^, ^34^, ^35^, ^36^ Furthermore, a comparison among the four viral strains revealed that Proto infection induced the strongest early immune responses in PBMCs, followed by Alpha, Delta, and Beta (Figures 4 and S1), which may be related to their pathogenicity.

Platelets are involved in the pathogenesis of COVID‐19, possibly increasing the risk of thrombosis.^37^ Furthermore, MKs, platelet precursors, have recently been identified as ACE2‐independent targets of SARS‐CoV‐2.^38^ SARS‐CoV‐2 infection of MKs and platelets in pulmonary tissues is associated with fatal COVID‐19. Clinical targeting of platelets may be a promising treatment to prevent viral spread, thrombus formation, and inflammation and increase survival.^39^ In the NHP model of this study, we found that genes enriched in negative regulation of MK differentiation were sharply upregulated in PBMCs at 3 and 5 dpi with Proto or Alpha (Figure 2C). Similarly, upregulated genes in the spleen were also enriched in this pathway (Figures 6C and S7). It will be interesting if a meta‐analysis is performed to examine the number of platelets in Proto‐infected patients with COVID‐19 or its variants.

Monocytes have frequently been reported to be associated with the severity of COVID‐19.^18^, ^40^, ^41^, ^42^, ^43^ In this study, we found that a long‐lasting response of monocytes was maintained in PBMCs of animals infected with Alpha or Beta strain but not with Proto and Delta strains, which is probably related to the pathogenicity of viruses (Figures 5C and S5A). *IFI27* has been recognized as a prognostic predictor for several diseases,^44^, ^45^ including COVID‐19.^46^ In this study, *IFI27* was highly upregulated in CD14+ cells of PMBC samples from animals infected with four strains of virus (Figure S5B), consistent with results from COVID‐19 patients. Cytokine storm is a detrimental factor in the pathogenesis of severe COVID‐19.^12^ Therefore, a balance of inflammatory and immune responses is critical for treating COVID‐19.^47^ IL‐15 is an immunoregulatory cytokine that modulates survival, proliferation, and function of immune cells, including NK cells, memory CD8+ T cells, and NKT cells.^48^ In memory B cells of this study, the IL‐15 receptor IL‐15RA was upregulated at 1 dpi with SARS‐CoV‐2 or its variants and then decreased at 3 and 5 dpi, indicating immunoregulation during SARS‐CoV‐2 infection (Figure S5C). IL‐1 is the key player in the cytokine storm of COVID‐19.^49^ In the NHP model of this study, *IL1b* in CD14+ cells were always at a very low level, which probably explains why the rhesus monkey in this model did not show severe symptoms.

Delta variant infection shows high risks of causing severe disease or death, the risk of which caused by the later pandemic Omicron variant is 0.72 of Delta variant.^50^ Infection of Delta resulted in activation of B cells, indicating production of antibody and initiation of immune response.^51^ Downregulated genes by Delta variant infection mainly belong to RPL and RPS family. SARS‐CoV‐2 infection interfered cellular function and metabolic procedures. Protein synthesis is inhibited by viral infection, leading to damage of immune system and further inhibition of antiviral ability.^52^ Downregulation of unique gene family RPL and RPS in Delta variant‐infected monkeys may explain the high risk of severe disease or death in Delta patients, the mechanism of which needs to be further studied. High expression of FCRL5 was observed in B memory cells of Alpha or Delta‐infected animals at 1 dpi, compared with those in Proto or Beta‐infected animals. Consistently, in a pediatric COVID‐19 patient who has severe symptoms and encephalopathy, FCRL5 is also highly expressed in tissue‐like B cells.^53^ The COVID‐19 patient comes from Beijing where Delta variant is circulating at that time. In another clinical study, nonsevere COVID‐19 patients show higher expression level of FCRL5 in spike‐specific B cells and stronger B cell response, as compared with severe COVID‐19 patients.^54^ The variants of Alpha and Delta are more prone to activation of B cells and production of memory cells, which may be beneficial to nonsevere COVID‐19 patients.

The immune characteristics of the rhesus monkeys infected by four strains show minimal differences in the early stages. Many of these differences are only in terms of their degree. For example, the type I interferon signaling pathway, ISG15‐protein conjugation defense response to bacterium, and cellular response to interferon‐alpha show more significant and sustained in Alpha, Beta, and Delta than Proto. This suggests that although the SARS‐CoV‐2 continuously produces new mutant strains, the initial antiviral pathways, such as interferon, remain effective against these new mutant strains. The upregulation of COVID‐19‐related genes was observed in the challenge of the four strains, and these findings were further supported by validation experiments using samples from a biobank. These results indicate that these genes, along with the pathways they are involved in, may serve as potential targets for the treatment of infections caused by new mutants, such as the XBB.1 and Eg.5 variants.^55^, ^56^

In order to accurately identify similarity and difference among SARS‐CoV‐2 Proto and variants, we performed single‐cell sequencing analysis of PBMCs from NHP under controlled conditions, including animals, infection dose and viral strains, which is the advantage of this study in the NHP model over clinical study of COVID‐19 patient. However, we did not have the variant Omicron at the time when we conducted the challenge experiment and single‐cell sequencing although Omicron began to become pandemic. Therefore, PBMCs from Omicron‐infected animal was not included in single‐cell sequencing. Later, we got one PBMC RNA sample of Omicron‐infected animal from biobank. The upregulation of COVID‐19‐related genes were analyzed in PBMCs of Omicron via RT‐PCR, results of which is consistent with those in PBMCs of Proto, Alpha, Beta, and Delta. However, it will be interesting if single‐cell sequencing of PBMCs from Omicron‐infected animal could be performed and compared with those in the other viral strains. Furthermore, we found some consistent results in this study with reported single‐cell sequencing analysis of PBMCs from COVID‐19 patients, including the main responder monocyte, which makes up for the defect in sample size of this study to some certain extent. The other limitation of this study is that there was only one animal for each group. However, we dynamically examined immune responses at 0, 1, 3, and 5 dpi. Thus, we could compare the immune status among all time points of each viral strain longitudinally, comprehensively revealing the dynamic immune responses to SARS‐CoV‐2 infection. However, more animals are needed to confirm the results of this study.

In conclusion, this study reveals the distinct early immune response of PMBCs postinfection with SARS‐CoV‐2 Proto or its variants at the single‐cell level, which provides important information for the precise prevention and treatment of COVID‐19.

## 4 MATERIALS AND METHODS

### 4.1 Biosafety

The animals was operated based on the guidelines of National Animal Research Authority (China) and the Experimental Animal Ethics Committee of the Institute in the ABSL‐3 facility of the National Kunming High‐level Biosafety Primate Research Center, Yunnan, China.

### 4.2 Animal ethics

All animal procedures were approved by the Institutional Animal Care and Use Committee of the Institute of Medical Biology, Chinese Academy of Medical Science (Ethics No. DWSP202101 001). The animal was housed with free access to food and water in negative‐pressure cages (one animal/cage) under controlled conditions (12‐h daylight cycle with light off at 8 PM).

### 4.3 Animal experiments

Four male rhesus monkeys (7–11 kg and 7−13 years old) were used for this study (Table S1). Animals were provided by the Kunming Primate Center of the Chinese Academy of Medical Sciences (Laboratory Animal Production License No. #SCXK (Dian)‐K2020‐0005). Rhesus monkeys were inoculated with prototype, Alpha, Beta, or Delta SARS‐CoV‐2 strains via intranasal (0.5 mL) and intratracheal (0.5 mL) routes at 10^7^ pfu/mL doses. The animals were monitored daily postviral inoculation. Blood samples (3 mL) and nasal swabs were collected at 0, 1, 3, and 5 dpi. All animals were sacrificed and dissected for collection of spleen and lung samples at 5 dpi.

### 4.4 Measurement of viral load

The viral load was determined by measuring the viral genomic *N* gene (F: GACCCCAAAATCAGCGAAAT, R: TCTGGTTACTGCCAGTTGAATCTG, probe: FAM‐ACCCCGCATTACGTTTGGTGGACC‐BHQ1).

### 4.5 RT‐qPCR

TRI Reagent (Sigma) and cDNA synthesis kit (Novoprotein; E047‐01A) was used to extract and reversely transcribe total RNA, respectively. The qPCR was performed on the cDNA using SYBR green (Novoprotein; E166‐01A) and the CFX384 Touch Real‐Time PCR Detection System (Bio‐Rad), and the −ΔΔCt method with GAPDH as reference. The RT‐qPCR primers used in this research is shown in Table S2.

### 4.6 Evaluation of inflammatory cytokines in serum

The multiplex assay was performed to determine inflammatory cytokine concentrations in serum samples by using the MILLIPLEX MAP NonHuman Primate Cytokine Magnetic Bead Panel‐Immunology Multiplex Assay.

### 4.7 Isolation of PBMCs

PBMCs were prepared from whole blood (5 mL) anticoagulant using the Ficoll‐Paque TM Plus kit (Cat#17144003; Cytiva) and according to the manufacturer's protocol.

### 4.8 Single‐cell RNA sequencing and analysis

PBMCs, isolated from EDTA‐anticoagulated blood from each animal via Ficoll‐Paque medium, were used for scRNA sequencing. The method and data processing procedure refer to the previous articles published by our laboratory. The Mmul10 *Macaca mulatta* was used as reference genome.

### 4.9 Transcriptomic analysis

The tissue sample (100 mg) was homogenized in 900 μL of TRIzol (Cat# 15596018; Invitrogen). In particular, the Mmul10 *Macaca mulatta* was used as reference genome and the dataset SRX858064 from GEO was used for experiment control.

### 4.10 Statistical analysis

Each figure legend provides details about the specific statistical analysis. Figures were generated using Adobe Illustrator, as well as the R packages ggplot2 and GraphPad Prism 8.

## AUTHOR CONTRIBUTION

Shuaiyao Lu, Xiaozhong Peng, Hongqi Liu, and Qiangming Sun contributed to the conception of the study; Wenhai Yu, Yanan Zhou, Chengyun Yang, Dexuan Kuang, Junbin Wang, Cong Tang, Haixuan Wang, Yuan Zhao, Hao Yang, Qing Huang, Daoju Wu, Yun Yang, Tingfu Du, and Bai Li performed the experiment; Hongqi Liu, Shuaiyao Lu, and Yun Yang contributed significantly to analysis and manuscript preparation; Hongqi Liu, Shuaiyao Lu, and Yun Yang performed the data analyses and wrote the manuscript; Hongqi Liu, Shuaiyao Lu, Tingfu Du, Wenhai Yu, Yanan Zhou, Hao Yang, Qing Huang, Daoju Wu, and Bai Li helped perform the analysis with constructive discussions. All authors have read and approved the final manuscript.

## CONFLICT OF INTEREST STATEMENT

All authors declared no potential conflict of interest.

## ETHICS STATEMENT

All animal procedures were approved by the Institutional Animal Care and Use Committee of the Institute of Medical Biology, Chinese Academy of Medical Science (Ethics No. DWSP202101 001).

## Supporting information

Supporting Information

Table S1

Table S2

## Data Availability

Raw sequencing data have been uploaded to the Sequence Read Archive (SRA) database, BioProject accession number PRJNA913633 contains scRNA‐seq data from PBMCs of SARS‐CoV‐2 Proto‐, Alpha‐, Beta‐, and Delta‐infected monkeys at dpi 0, 1, 3, 5, and accession number PRJNA910844 contains mRNA‐seq sequencing data from spleens at dpi 5. All data will be released in December 2023.
