# Supplementary material for: Single‐cell transcriptomic atlas of distinct early immune responses induced by SARS‐CoV‐2 Proto or its variants in rhesus monkey
Source: MedComm (2020). 2023 Nov 20;4(6):e432. doi: 10.1002/mco2.432 (PMC10661830; doi:10.1002/mco2.432)
Supplement: Supplementary file 1 — Supporting Information [file MCO2-4-e432-s001.docx]

**Supplementary Information**

**Single-cell transcriptomic atlas of distinct early immune responses induced by SARS-CoV-2 Proto or its variants in rhesus monkey**

**Yun Yang** ^1^^#^, **Tingfu Du**^1#^, **Wenhai Yu**^1#^, **Yanan Zhou**^1#^, **Chengyun Yang**^1#^, Dexuan Kuang^1^, Junbin Wang^1^, Cong Tang^1^, Haixuan Wang^1^, Yuan Zhao^1^, Hao Yang^1^, Qing Huang^1^, Daoju Wu^1^, Bai Li^1^, Qiangming Sun^1,3*^,Hongqi Liu^1,3*^, Shuaiyao Lu^1,3*^, Xiaozhong Peng^1,2,3*^

1. Institute of Medical Biology, Chinese Academy of Medical Sciences and Peking Union Medical School, Kunming, China 650118;

2. State Key Laboratory of Medical Molecular Biology, Department of Molecular Biology and Biochemistry, Institute of Basic Medical Sciences, Medical Primate Research Center, Neuroscience Center, Chinese Academy of Medical Sciences, School of Basic Medicine, Peking Union Medical College, Beijing, China 100051;

3. Key Laboratory of Pathogen Infection Prevention and Control (Peking Union Medical College), Ministry of Education, Beijing, China

**Running title:** Different early immune responses induced by SARS-CoV-2

# These authors contribute equally: **Yun Yang**, **Tingfu Du**, **Wenhai Yu**, **Yanan Zhou**, **Chengyun Yang**

***Corresponding author:**

Qiangming Sun , [qsun@imbcams.com.cn](mailto:qsun@imbcams.com.cn)

Hongqi Liu , [lhq@Imbcams.com.cn](mailto:lhq@Imbcams.com.cn)

Shuaiyao Lu, [lushuaiyao-km@163.com](mailto:lushuaiyao-km@163.com)

Xiaozhong Peng, [pengxiaozhong@pumc.edu.cn](mailto:pengxiaozhong@pumc.edu.cn)

**Content**

**Supplemental Figures**

**Figure S1** Characterization of SARS-CoV-2 infection in rhesus monkeys

**Figure S2** mRNA levels of COVID-19 related genes in PBMCs part I

**Figure S3** mRNA levels of COVID-19 related genes in PBMCs part II

**Figure S4** mRNA levels of COVID-19 related genes in PBMCs part III

**Figure S5** CD16+ monocytes, B memory cells and inflammatory in PBMCs long-standing responses

**Figure S6** Expressions of COVID-19 related genes in NK, CD8+ T, CD4+ T central memory cells

**Figure S7** Major GO pathways (p < 0.05) enriched with upregulated or downregulated genes in the spleen of Proto, Alpha, Beta, and Delta strain.

**Supplemental Tables**

**Table S1** Characteristic information of monkeys

**Table S2** Primers used in RT-qPCR


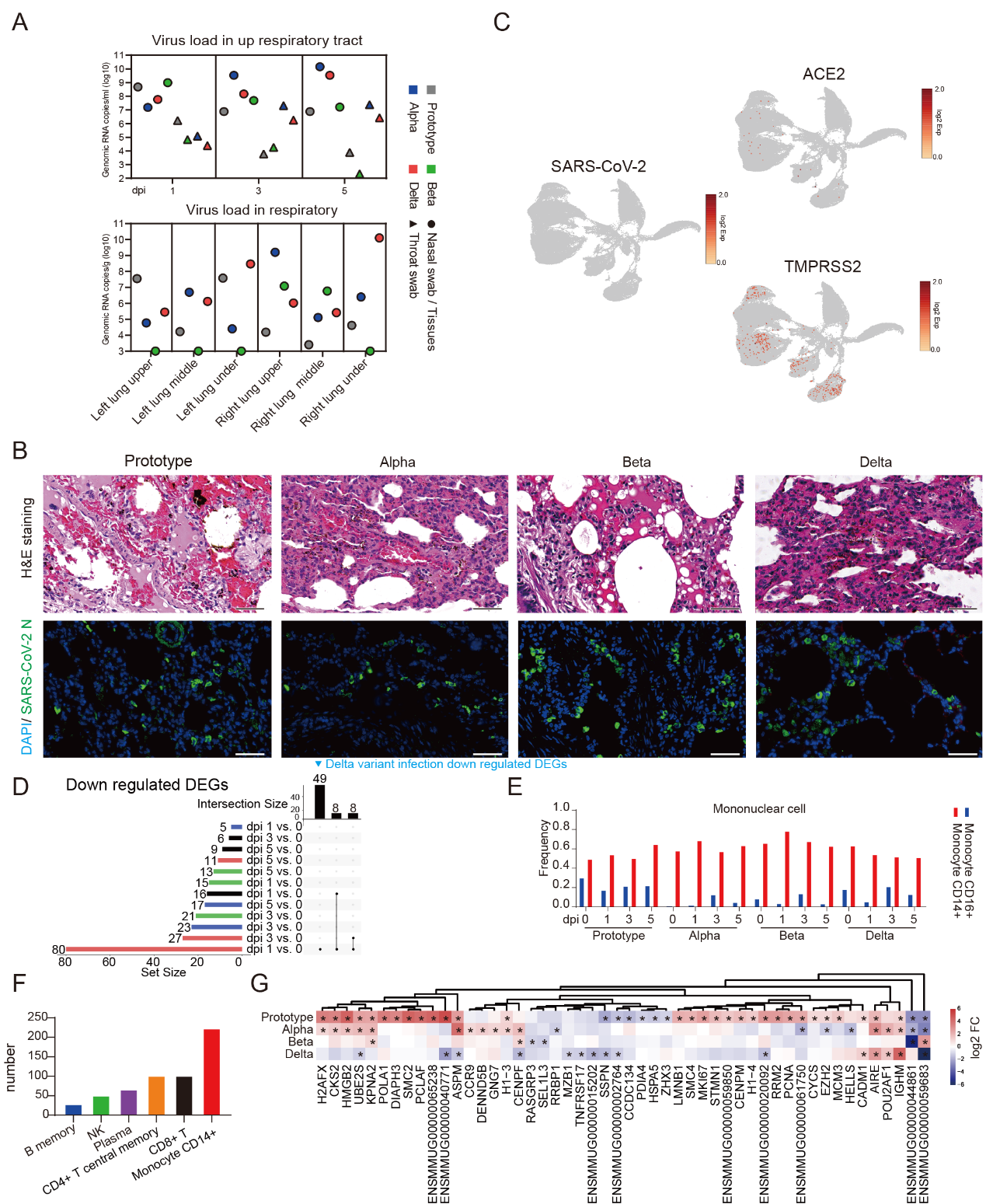


**Supplementary Figure 1** Characterization of SARS-CoV-2 infection in rhesus monkeys. A. Viral loads were determined in nasal swab (dot) and throat swab (triangle) in the left panel, and lung tissues in the right panel by qRT-PCR on 5 dpi (Prototype-gray, Alpha-blue, Beta-green and Delta-red). B. Hematoxylin and eosin (H&E) staining (up) and Immunofluorescence (IF) staining of SARS-CoV-2 N protein (down) in the infected lungs (the bar length represents 50 μm). C. Heatmaps for mRNA expression level of *ACE2*, *TMPRSS2* and viral RNA in PBMCs via UMAP. D. UpSet plots of integral down-regulated DEGs of PBMCs collected at 1, 3, or 5 dpi vs. 0 dpi. The set size bar is colored by variants: prototype (gray), Alpha (blue), Beta (green), and Delta (red). E. Frequencies of mononuclear cell in PBMCs collected at 0, 1, 3, and 5 dpi. F. Distribution of DEGs in cell lineages of PBMCs collected at 1 dpi vs. 0 dpi (p < 0.05). G: DGEs in plasma cells of PBMCs at 1 dpi (vs. 0 dpi) (p < 0.05).

**
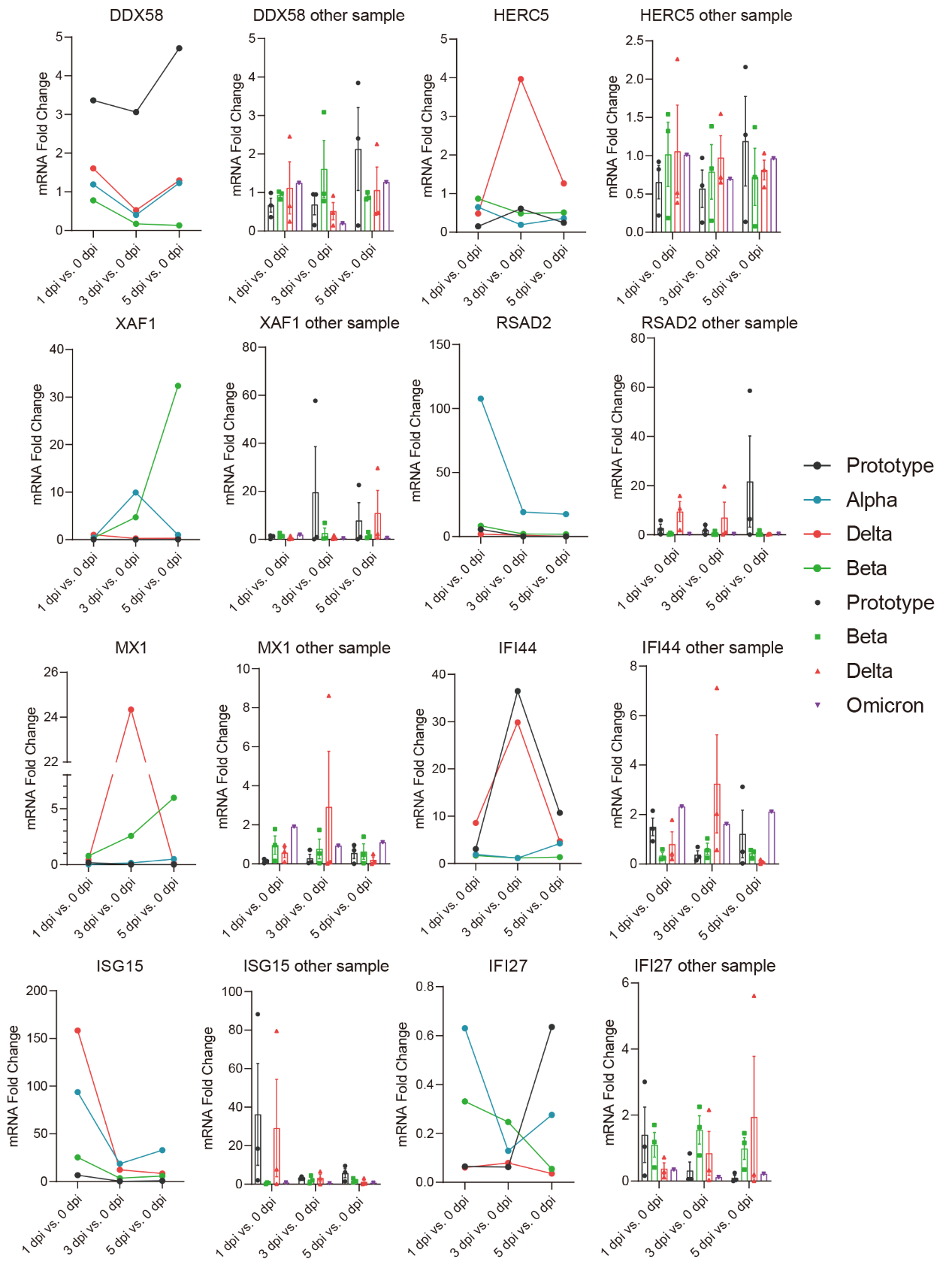
**

**Supplementary Figure 2** mRNA levels of COVID-19 related genes in PBMCs part I**.** COVID-19 related genes (Fig2D, *DDX58*, *HERC5, XAF1, RSAD2, MX1, IFI44, ISG15, IFI27*) at mRNA level of PBMCs in four monkeys infected in this study (Dotted line graphs) as well as other rhesus monkey samples (prototype, n=3; beta, n=3; delta, n=3; omicron, n=1) from biobank (Bar graphs).


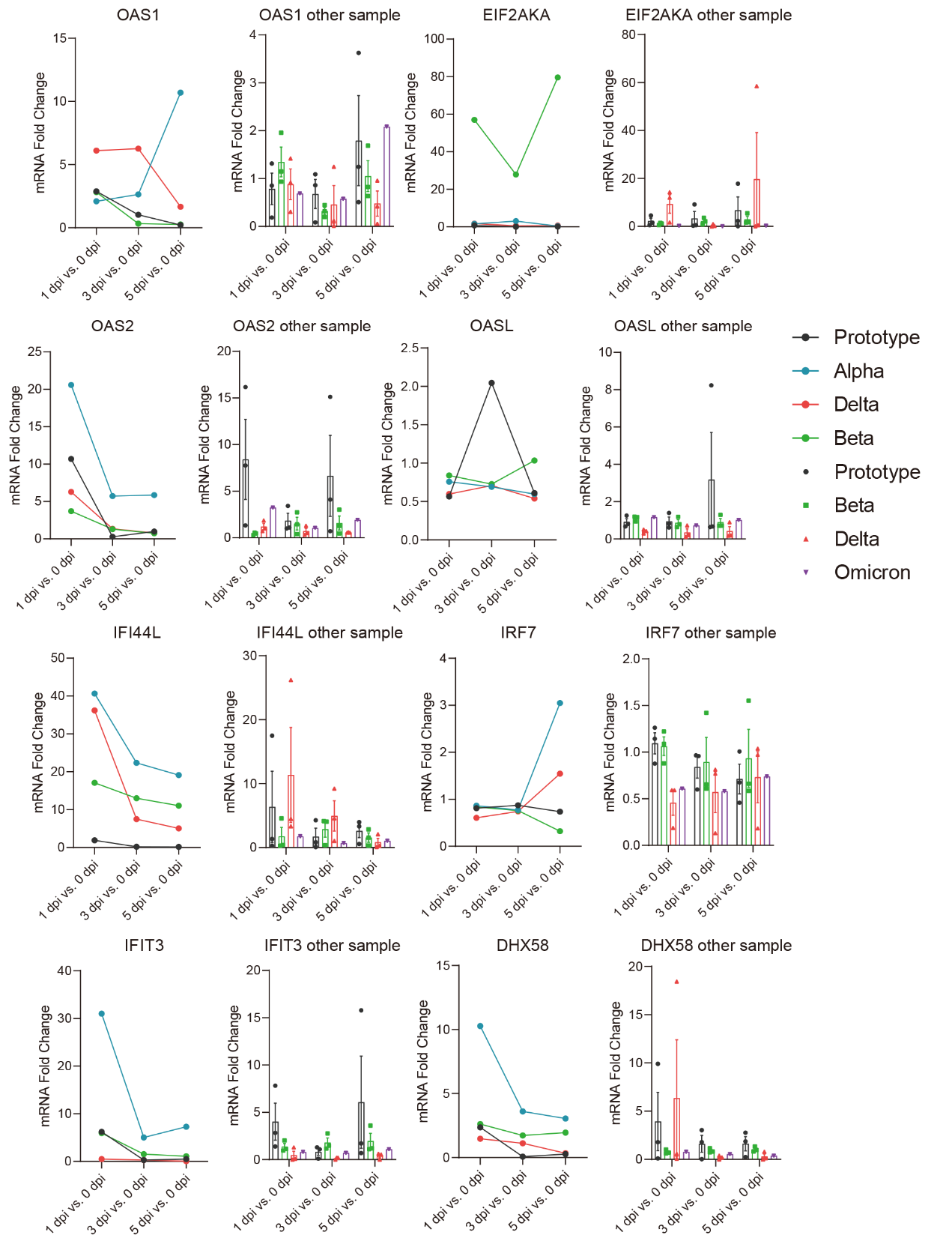


**Supplementary Figure 3** mRNA levels of COVID-19 related genes in PBMCs part II. COVID-19 related genes (Fig2D, *OAS1, EIF2AKA, OAS2, OASL, IFI44L, IRF7, IFIT3, DHX58*) at mRNA level of PBMCs in four monkeys infected in this study (Dotted line graphs) as well as other rhesus monkey samples (prototype, n=3; beta, n=3; delta, n=3; omicron, n=1) from biobank (Bar graphs).


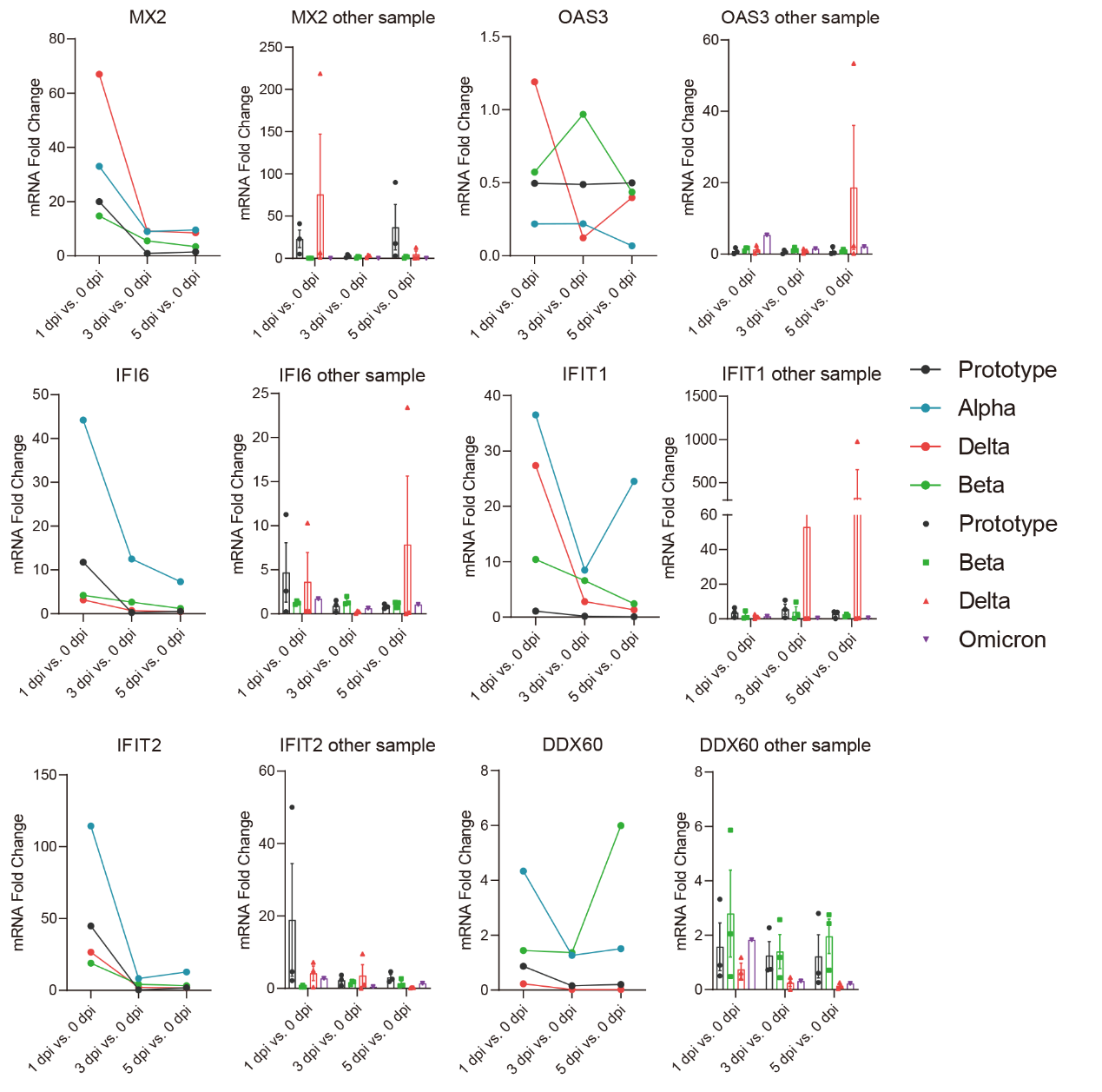


**Supplementary Figure 4** mRNA levels of COVID-19 related genes in PBMCs part III. COVID-19 related genes (Fig2D, *MX2, OAS3, IFI6, IFIT1, IFIT2, DDX60*) at mRNA level of PBMCs in four monkeys infected in this study (Dotted line graphs) as well as other rhesus monkey samples (prototype, n=3; beta, n=3; delta, n=3; omicron, n=1) from biobank (Bar graphs).


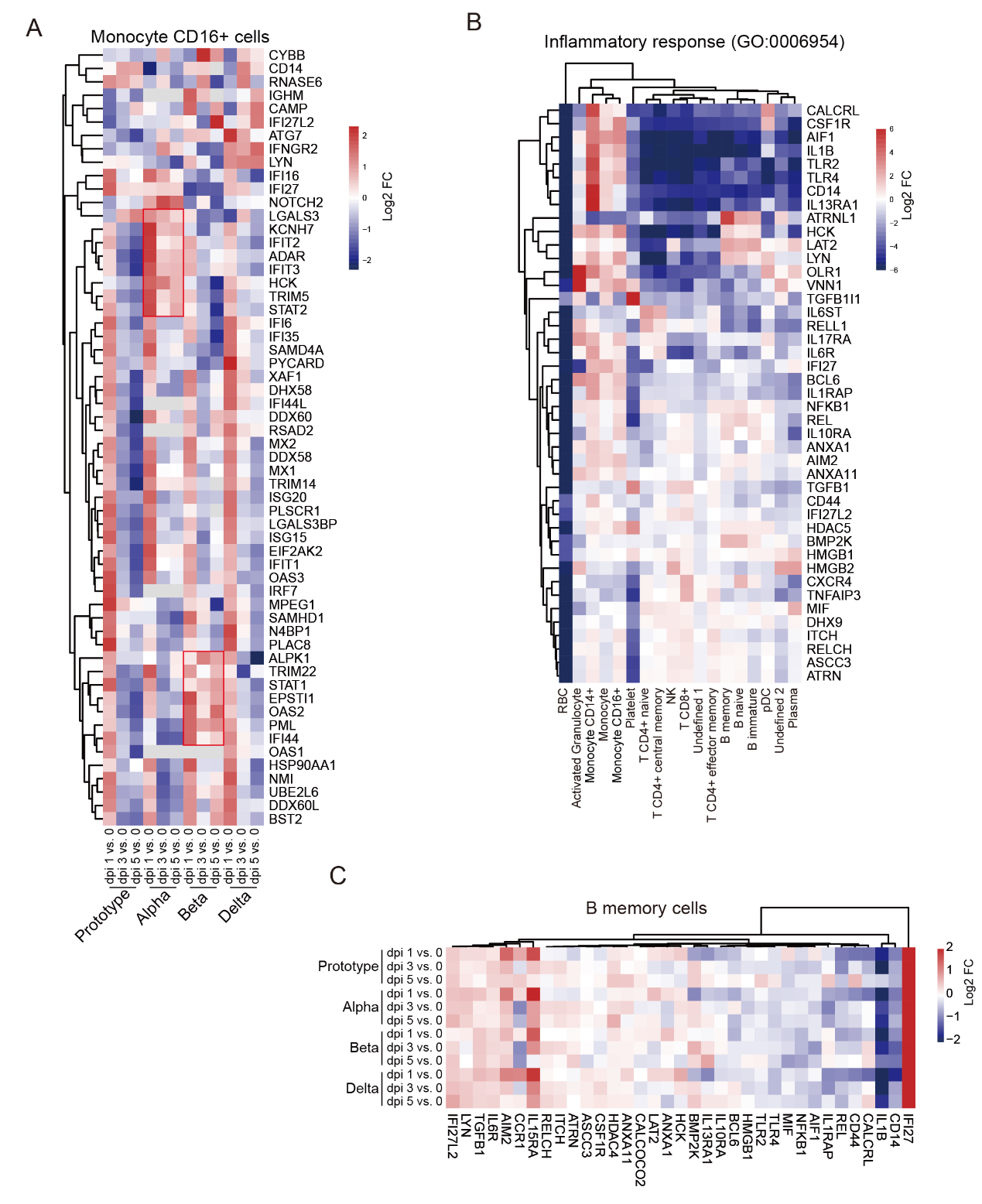


**Supplementary Figure 5** CD16+ monocytes, B memory cells and inflammatory in PBMCs long-standing responses. A. Expression levels of COVID-19-related genes in CD16+ cells of PBMCs collected at 1, 3, and 5 dpi (vs. 0 dpi). B. Expression levels of genes involved in the inflammatory response (GO: 0006954) in a subpopulation of PBMCs. C. Expression levels of COVID-19-related genes in B memory cells of PBMCs collected at 1, 3, and 5 dpi (vs. 0 dpi)


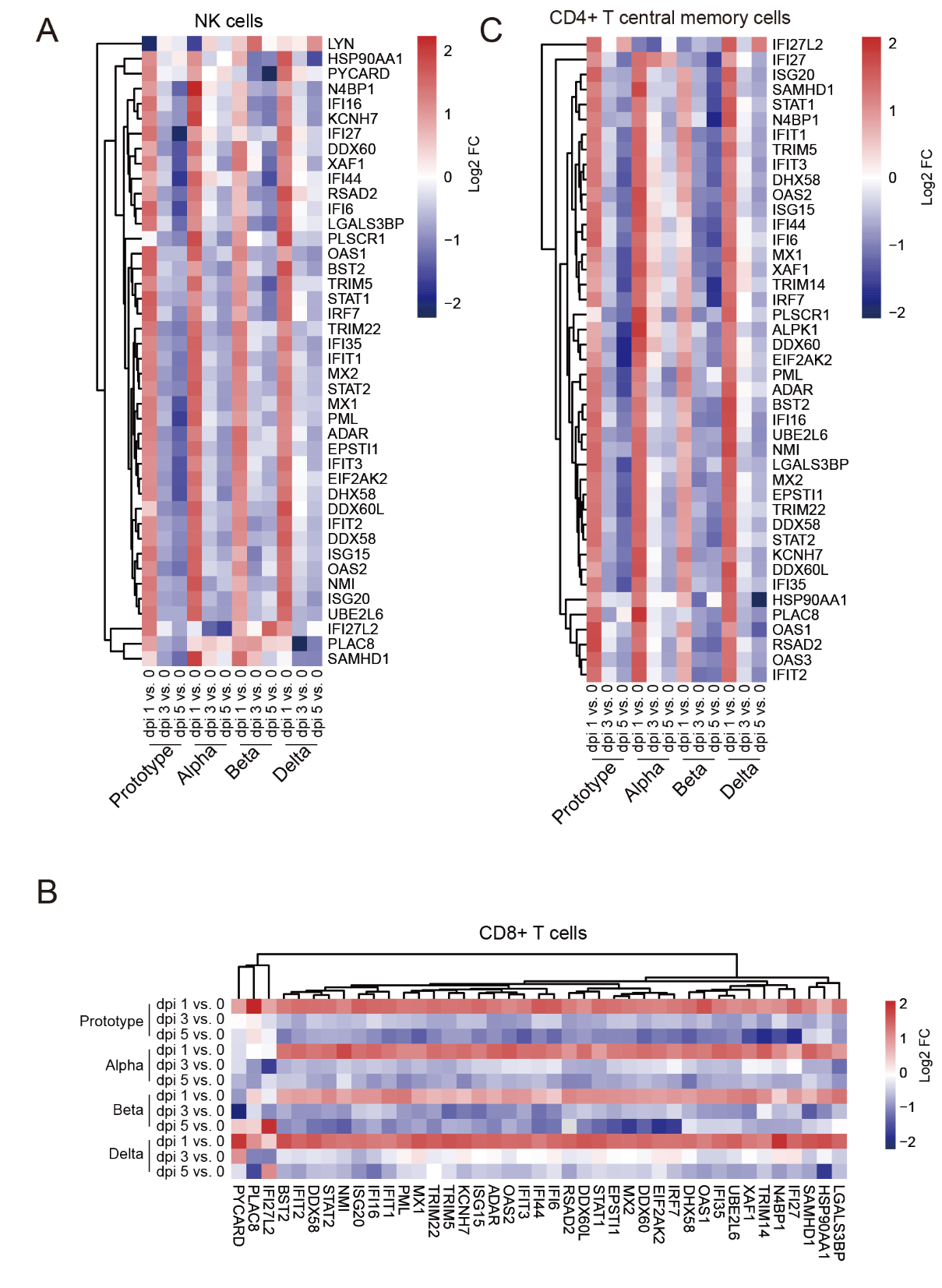


**Supplementary Figure 6** Expressions of COVID-19 related genes in NK, CD8+ T, CD4+ T central memory cells. Heatmaps shows DEGs of COVID-19 related genes (post-infection vs dpi 0) in the indicated cell populations (A. NK cells; B. CD4+ T central memory cells; C. CD8+ T cells) of PBMCs at days 1, 3, and 5 post variant infections.


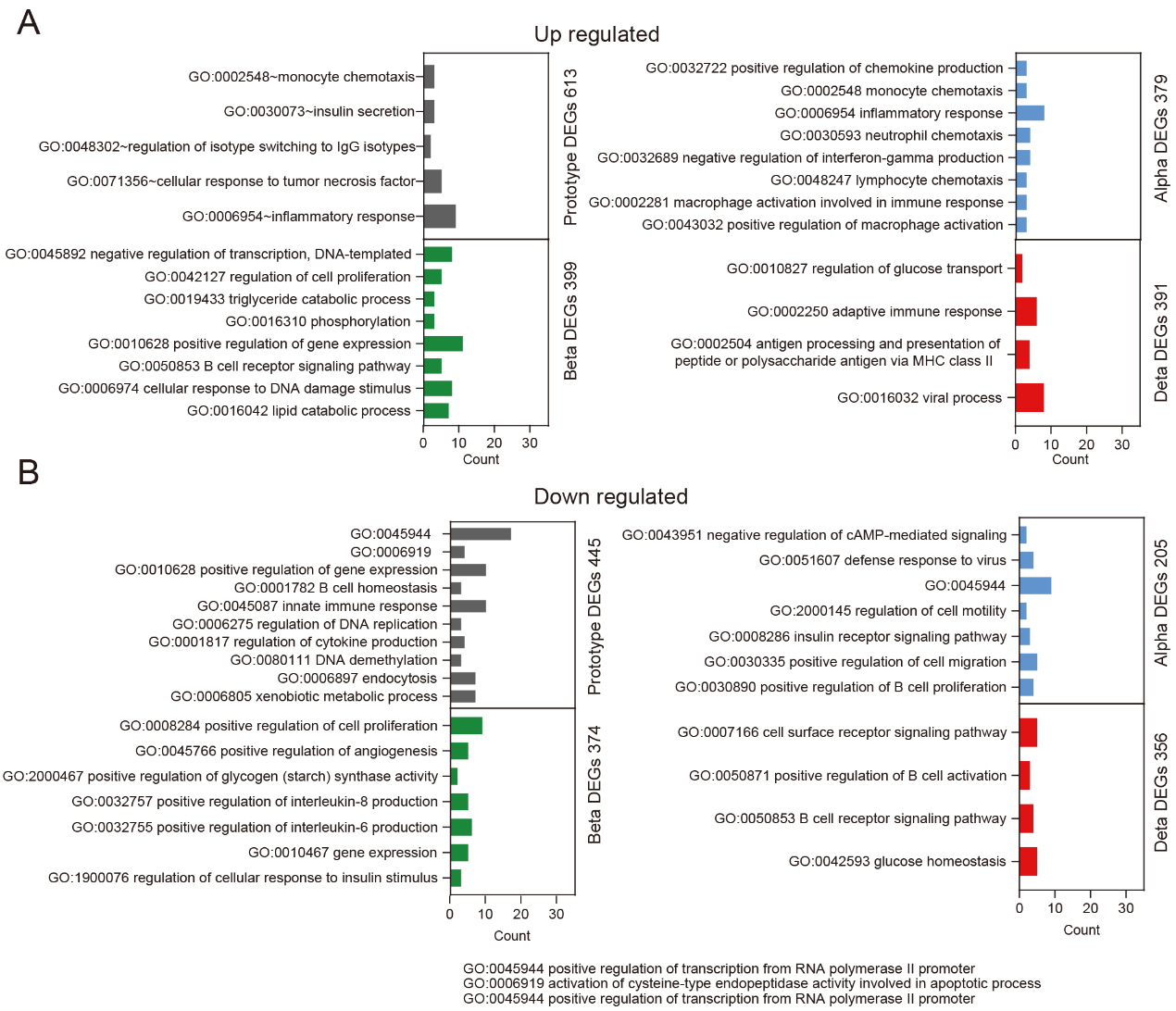


**Supplementary Figure 7** Major GO pathways (p < 0.05) enriched with upregulated or downregulated genes in the spleen of Proto, Alpha, Beta, and Delta strain. A. Gene Ontology (GO) analysis on the distinctively up-regulated genes within groups Proto, Alpha, Beta, and Delta. B. GO analysis on the distinctively down-regulated genes within groups Proto, Alpha, Beta, and Delta.

**Supplementary Table 1** Characteristic information of monkeys

| Animal ID | Gender | Age(years old) at the time of euthanasia | Equivalent age range in humans (year) | The infected strain |
| --- | --- | --- | --- | --- |
| 08041 | Male | 13 | 39 | Prototype |
| 11249 | Male | 10 | 30 | Alpha |
| 11263 | Male | 10 | 30 | Delta |
| 1412079 | Male | 7 | 21 | Beta |

**Supplementary Table 2** Primers used in RT-qPCR

| Gene name | Forward primers (5'-3') | Reverse primers (5'-3') |
| --- | --- | --- |
| *DDX58* | TGTGCTCCTACAGGTTGTGGA | TACTGGGATCTGATTTGCAAAA |
| *DDX60* | CAGCGCCAATGAAATGGTGCC | CTCAGGGGTTTATGAGAATGCC |
| *DHX58* | GCCCCCGGGGTATCATCTTC | CCCGAATGTCCACAGTCTG |
| *EIF2AK2* | GCTAGAAGTGACAGCCCAGTG | ATACCCGGTGTTCCTCAAATA |
| *EPSTI1* | AAGTGGAAGGAGCAGAACAGAG | GCTTTTGCTGGTATTTAGATTGC |
| *HERC5* | GGTGAGCTTTTTGCCTGGG | TTCTCCGGCAGAAATCTGAGC |
| *IFI27* | CGCTTTCACCTCATCAGCAGT | CACAACTCCTCCAATCACGACT |
| *IFI44* | ATGGCAGTGACAACTCGTTTG | TCCTGGTAACTCTTTTCTGCATA |
| *IFI44L* | AGTTGCCTTGATTCTGACATT | TATCTTAGCCCACTGCTTCTC |
| *IFI6* | GGTGGAGGCAGGCAAGAAAGAGTG | GCAGACCAGCTCATCAGCGAGGC |
| *IFIT1* | GCACTGGGTATGCGATCTC | CAGCCTGCCTTAGGGGAAG |
| *IFIT2* | GACACGGTTAAAGTGTGGAGG | TCCAGACGGTAGCTTGCTATT |
| *IFIT3* | AGAAAAGGTGACCTAGACAAAGC | CCTTGTAGCAGCACCCAATCT |
| *IRF7* | GCTGGACGTGACCATCATGTA | GGGCCGTACAGGAACATGC |
| *ISG15* | TGGACAAGTGCGATGAACCTC | TCAGCTGTACCTCGTAGGTG |
| *MX1* | AGTGGGATCGTGACTAGAT | TGACCTTGCCTCTCCACTCATC |
| *MX2* | CAGAGGCAGCGGAATCGTAA | TGCAGCTCTATCTCGGTGTTC |
| *OAS1* | TGTCCAAGGTGGTAAAGGGTG | CCGGCGATTTAACTGATCCTG |
| *OAS2* | ACATGACATCCTCGATAAAACTG | GAACCCATCATGGGACTTCTG |
| *OAS3* | GAAGGAGTTCACAGAGACGGCG | CCCTTGACGATTTTCAGCACC |
| *OASL* | CCATCGTGCCTGCCTACAGAG | CTTCAGCTTAGTTGGCCGATG |
| *RSAD2* | TTGGATATTCTCGCTGTCTCCT | AGTGCTTTGATCTGTTCCGTC |
| *SAMD4A* | TCGAGGCTTTGGGCAGTCC | GAGCTGACGAATCCACTGGT |
| *XAF1* | CTCACTGCCTGCGGTTCCTGGTCT | TGGCACTCATTGGCCTGCTGGTG |
| *GAPDH* | ACAACTTTGGTATCGTGGAAGG | GCCATCACGCCACAGTTTC |
